# Temperature-Responsive Nano-Biomaterials from Genetically Encoded Farnesylated Disordered Proteins

**DOI:** 10.1021/acsabm.1c01162

**Published:** 2022-01-19

**Authors:** Md. Shahadat Hossain, Zhe Zhang, Sudhat Ashok, Ashley R. Jenks, Christopher J. Lynch, James L. Hougland, Davoud Mozhdehi

**Affiliations:** † Department of Chemistry, Syracuse University, Syracuse, New York 13244, United States; ‡ Department of Biology, Syracuse University, Syracuse, New York 13244, United States; § Department of Biomedical and Chemical Engineering, Syracuse University, Syracuse, New York 13244, United States; ∥ BioInspired Syracuse: Institute for Material and Living Systems, Syracuse University, Syracuse, New York 13244, United States

**Keywords:** farnesylation, lipidated biopolymers, recombinant nano-biomaterials, post-translational modification, lipidation, self-assembly, bioconjugation

## Abstract

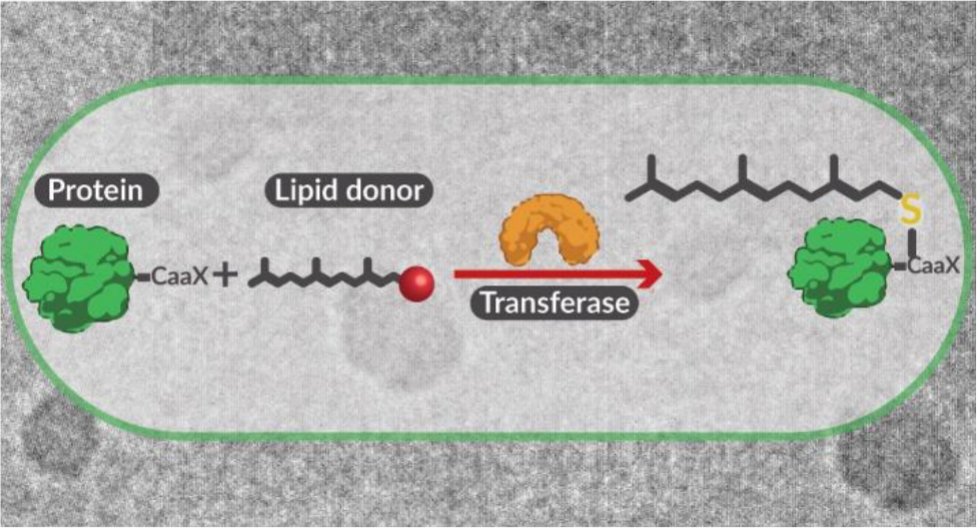

Despite broad interest in understanding the biological implications of protein farnesylation in regulating different facets of cell biology, the use of this post-translational modification to develop protein-based materials and therapies remains underexplored. The progress has been slow due to the lack of accessible methodologies to generate farnesylated proteins with broad physicochemical diversities rapidly. This limitation, in turn, has hindered the empirical elucidation of farnesylated proteins’ sequence–structure–function rules. To address this gap, we genetically engineered prokaryotes to develop operationally simple, high-yield biosynthetic routes to produce farnesylated proteins and revealed determinants of their emergent material properties (nano-aggregation and phase-behavior) using scattering, calorimetry, and microscopy. These outcomes foster the development of farnesylated proteins as recombinant therapeutics or biomaterials with molecularly programmable assembly.

## Introduction

The development of new protein-based biopharmaceuticals and materials is a vibrant area of research at the interface of chemistry, biology, and materials science and engineering.^1−8^ Efforts in this space have traditionally focused on engineering the amino acid sequence and structure of proteins to achieve a specific function for applications including biomaterials,^9−12^ sensors,^13^ and biocatalysts,^14^ among others.^15−17^ Instead of changing the sequence of proteins, cells leverage an alternative strategy, post-translational modification (PTMs), to modulate a protein’s structure and function with exquisite spatiotemporal control.^18^ Despite the significant interest in the biology of PTMs,^19^ this strategy remains underutilized for diversifying physicochemical properties, engineering capabilities, and the biological behavior of proteins.^20−23^ This lacuna exists because the synthesis of proteins with compositionally defined PTM patterns remains challenging, and although more than 300 PTMs have been identified, the energetic interplay and (bio)material consequences of only a handful of these modifications have been systematically studied.^24^

Lipidation is a common PTM in eukaryotes where the nature of the attached lipid (e.g., fatty acids, sterols, isoprenoids, etc.) dictates biological outcomes by controlling protein structure, function, and localization.^25^ For instance, isoprenylation of Ras signaling proteins—the modification of cysteine residues with either 15 carbon (farnesyl) or a 20 carbon (geranylgeranyl) isoprenoid lipid—is critical for their localization to correct membrane-bound organelles, as well as regulating their function.^26^ While the biology of isoprenylation and the interactions between prenylated proteins and lipid bilayer are of broad research interest,^27−30^ application of this PTM to control the assembly and function of protein-based materials remains underexplored.

This is mainly due to the lack of a facile, scalable, and inexpensive synthetic methods to modify proteins with lipids such as isoprenoids. Many lipidated proteins cannot be produced by genetic code expansion methods due to the stringent preference of ribosomes for amino acid-derived motifs.^31^ Alternatively, their multistep convergent semisynthesis is laborious and technically challenging.^32^ The inability to rapidly generate lipid-modified proteins has hindered the empirical elucidation of their sequence–structure–function rules and the computational parametrization of their design space.^33^

**Figure 1:**
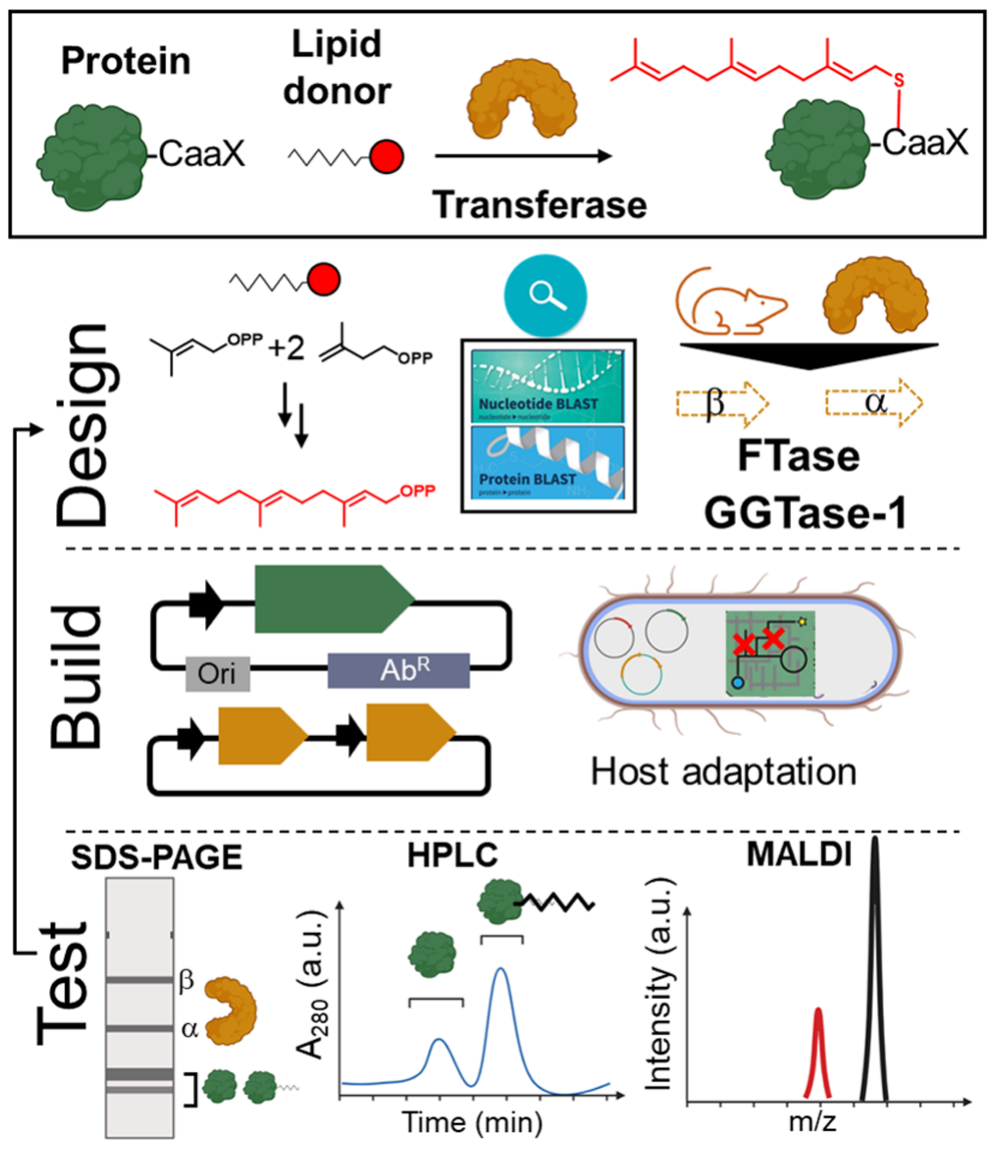
Schematic of our three-prong approach to engineer recombinant farnesylation platforms. *Design*. Identify missing enzymes required for the synthesis or transfer of lipid donors in *E. coli* and select isofunctional enzymes for recombinant expression in bacteria. *Build*. Clone genes encoding proteins into orthogonal plasmids for coexpression. *Test*. Use small-scale expression screening to identify conditions for high-yield heterologous production of proteins and lipidated products. The figure was created with BioRender.com.

To address this gap, we developed an operationally simple, high-yield biosynthetic route to produce prenylated proteins by genetically engineering *E. coli* to coexpress the desired protein and the minimum enzymatic machinery required for prenylation (Figure 1). In eukaryotes, isoprenylation is carried out by specialized transferases that bind to a lipid donor (isoprenyl pyrophosphate) and modify a recognized peptide substrate fused to the C-terminus of target proteins. Herein, we focused on farnesylation because *E. coli* endogenously biosynthesizes farnesyl pyrophosphate (FPP)—providing access to the lipid donor without the need for genetic/metabolic engineering.^34^

The next component of the minimal recombinant farnesylation system is a prenyltransferase enzyme that can be heterologously expressed in an active form in bacteria. Three classes of prenyltransferases are known: farnesyltransferase (FTase) and geranylgeranyltransferase (GGTase) types I and II.^35^ Here, we opted to investigate the potential utility of FTase and GGTase-I due to their broad substrate scopes: proteins fused to the “CaaX” motif at the C-terminus. In this motif, the cysteine, which is the site of PTM, is followed by two hydrophobic amino acids; and the final amino acid (X) determines preferential selectivity toward FTase (X = Ser) or GGTase-I (X= Leu).

To reveal how farnesylation modulates the assembly and properties of proteins, we focused on elastin-like polypeptides (ELPs) as a model system. ELPs are artificial peptide polymers derived from the consensus sequence of tropoelastin (GZGVP), in which the guest reside (Z) can be any amino acids other than proline.^36^ We chose ELP as a model system for two reasons: (1) ELPs have a well-characterized lower critical solubility transition (LCST) behavior in which they undergo a soluble-to-insoluble temperature above a critical transition temperature (*T*_t_). In addition to solution parameters^37^ (e.g., concentration or ionic strength), this *T*_t_ depends on the molecular syntax of the ELP^38^ (e.g., the hydrophobicity of the guest residue, the length of the polypeptide) and the physicochemistry of the PTM motif.^39−44^ The phase-separation behavior of ELPs can be conveniently monitored using various scattering techniques, and this, therefore, enables us to parse the effect of farnesylation on the thermoresponse of lipid–protein conjugates. (2) ELPs can be expressed at high yields in *E. coli* and purified at scale using nonchromatography techniques that leverage their reversible temperature-triggered phase behavior. Additionally, the stimuli-responsive characteristics of ELPs enable the fine-tuning of the emergent assembly of farnesylated protein with temperature.

The remainder of this paper is organized as follows: We first describe the construction of genetically modified strains for one-pot recombinant expression-lipidation of farnesyl-modified proteins. We then discuss the effects of farnesylation and its interplay with sequence hydrophobicity and length on emergent properties (the thermo-response, nano-, and mesoscale) of model lipidated proteins using complementary techniques, including turbidity, differential scanning calorimetry, dynamic light scattering, and microscopy.

## Experimental Section

### Cloning

Genes encoding α and β subunits of FTase and GGTase-I (from *Rattus norvegicus*) and their canonical substrate peptides (CVLS and CVLL) were constructed using standard molecular biology techniques. All cloning steps were conducted using *E. coli* EB5α strain. The identity of each gene was confirmed by Sanger sequencing. After verifications, plasmids were cotransformed to *E. coli* BL21(DE3) for protein expression. See the Supporting Information and Table S1 for additional details.

### Expression of Nonlipidated Constructs

A bacterial colony was used to inoculate a flask containing 50 mL of sterile 2x YT medium supplemented with kanamycin (45 μg/mL). After overnight growth in a shaking incubator (37 °C, 200 rpm, 16 h), 4 mL of this culture was used to inoculate 1 L of 2x YT media. The bacteria were grown in an orbital shaker incubator at 37 °C at 200 rpm. The expression was induced by adding isopropyl β-d-1-thiogalactopyranoside (IPTG) to a final concentration of 1 mM when the optical density reached 1.5. After 18 h, cells were harvested by centrifugation (3745*g*, 4 °C for 30 min). The cell pellets were resuspended in phosphate buffer saline (PBS, pH 7.4, 10 mL/L of expression culture) and stored at −80 °C until purification.

### One-Pot Expression–Lipidation of Farnesylated Proteins

A 50 mL portion of sterile 2x YT medium, supplemented with ampicillin (100 μg/mL) and chloramphenicol (25 μg/mL), was inoculated with a bacterial colony and incubated in an orbital shaker (37 °C, 200 rpm) to OD_600_ of 1.5. A 4 mL portion of this media was used to inoculate each one liter of 2x YT medium. The bacteria were grown in an orbital shaker incubator at 37 °C at 200 rpm. After reaching an OD_600_ of 0.8, the temperature was reduced to 28 °C, and the expression was induced by adding IPTG and ZnSO_4_ to the final concentrations of 1 and 0.5 mM, respectively. After continuing the expression at 28 °C for 18 h, the cells were harvested by centrifugation (3745*g*, 4 °C, 30 min) and subsequently resuspended in PBS (5 mL/L of expression culture).

### Protein Purification

To isolate post-translationally lipidated proteins, we adapted a recently reported method for organic extraction of ELPs from the cell pellet.^45^ Briefly, the cell pellet was incubated with isopropanol, which lyses the cells and precipitates most endogenous proteins and nucleic acids—while maintaining the solubility of expressed ELPs. After separating insoluble debris, ELPs are precipitated by adding acetonitrile (up to 70% (v/v)). The protein pellet was resuspended in ethanol:water, 50% (v/v), and purified by preparative RP-HPLC to ensure >95% purify for characterization studies. Additional details are provided in the Supporting Information.

### RP-HPLC

Preparative and analytical reverse phase high performance liquid chromatographies (RP-HPLCs) were performed on a Shimadzu instrument equipped with a photodiode array detector on C18 columns (Phenomenex Jupiter 5 μm C18 300 Å, 250 × 4.6 mm and 250 × 10 mm). A mobile phase consisting of a gradient of acetonitrile and water (supplemented with 0.1% TFA) was used to elute the proteins (Table S2).

### Turbidimetry Assay

The thermal response of proteins was analyzed using a Cary 100 UV–vis spectrophotometer (Agilent, Santa Clara, CA) equipped with a Peltier temperature controller. The absorbance of protein solutions at 350 nm was continuously monitored between 15 and 65 °C while heating/cooling the solution at the rate of 1 °C/min.

### Matrix-Assisted Laser Desorption/Ionization, Time-of-Flight Mass Spectrometry (MALDI-TOF-MS)

The molecular weight of proteins was determined using MALDI-TOF-MS, conducted on a Bruker microflex LRF instrument. To determine the location of the farnesyl group, the proteins were digested with trypsin, and the peptide fragments were analyzed using MALDI-TOF-MS.

### Multiangle Dynamic Light Scattering (MADLS)

MADLS was performed using Zetasizer Ultra (Malvern Instruments, UK) at the scattering angles of 13°, 90°, and 173°. Protein solutions (6 μM in PBS) were filtered into a DLS cuvette and analyzed at 15–65 °C at 5 °C increments. For each construct, smaller temperature increments (0.5–1 °C) were applied within ±5 °C of the transition temperature determined from turbidimetry experiments. Measurements were performed in triplicate after incubating the samples for 3 min at each temperature. Scattering autocorrelation functions were analyzed with Zetasizer software using cumulant and CONTIN methods to derive the average hydrodynamic radius (*Z*_avg_), polydispersity index, and the intensity-size distributions.

### Cryo-Transmission Electron Microscopy (cryo-TEM)

Protein solutions were applied to plasma-treated (Pelco easiGlow, negative polarity, 45 s, 30 mA) Quantifoil copper grids (Q3100CR1.3, Electron Microscopy Sciences, PA). After plunge freezing in liquid ethane, grids were imaged on a Tecnai BioTwin 120 kV transmission electron microscope. Images were collected on a Gatan SC1000A CCD camera and analyzed using ImageJ.

### Differential Scanning Calorimetry (DSC)

NanoDSC (TA Instruments, New Castle) was used to quantify the enthalpy of phase-separation by measuring the excess heat capacity of the protein solution (against PBS reference) while heating the sample, 10–65 °C at a rate of 1 °C/min.

### Attenuated Total Reflectance Fourier-Transform Infrared Spectroscopy (ATR-FTIR)

The FT-IR absorption spectra were collected using Thermo Scientific Nicolet iS5 FT-IR spectrometer with iD7 attenuated total reflectance accessory by sandwiching the lyophilized proteins directly over the crystal. The number of scans and spectral resolution were set to 128 and 4 cm^–1^, respectively.

### Proton Nuclear Magnetic Resonance (^1^H NMR)

^1^H NMR spectra were recorded on a Bruker Avance III HD 400 MHz. The samples were prepared by dissolving lyophilized proteins into deuterium oxide or dimethyl sulfoxide-*d*_6_ at the concentration of 1.67 mg/mL. The proton NMR spectra were collected at 25 °C.

### Differential Interference Contrast (DIC) Microscopy

DIC was performed with a Zeiss AxioObserver Z1 widefield microscope (Carl Zeiss Inc., Berlin, Germany) equipped with an ORCA-Flash4.0 LT+ Digital CMOS camera (Hamamatsu Photonics, Hamamatsu, Japan). Protein solutions (200 μM in PBS) were heated to 60 °C. The coacervates were imaged immediately after deposition onto a glass slide, shielded with a coverslip. Images were processed and analyzed using MetaMorph imaging software (Molecular Devices, CA) and ImageJ.

## Results and Discussion

### Recombinant Production of Farnesylated Proteins

**Figure 2:**
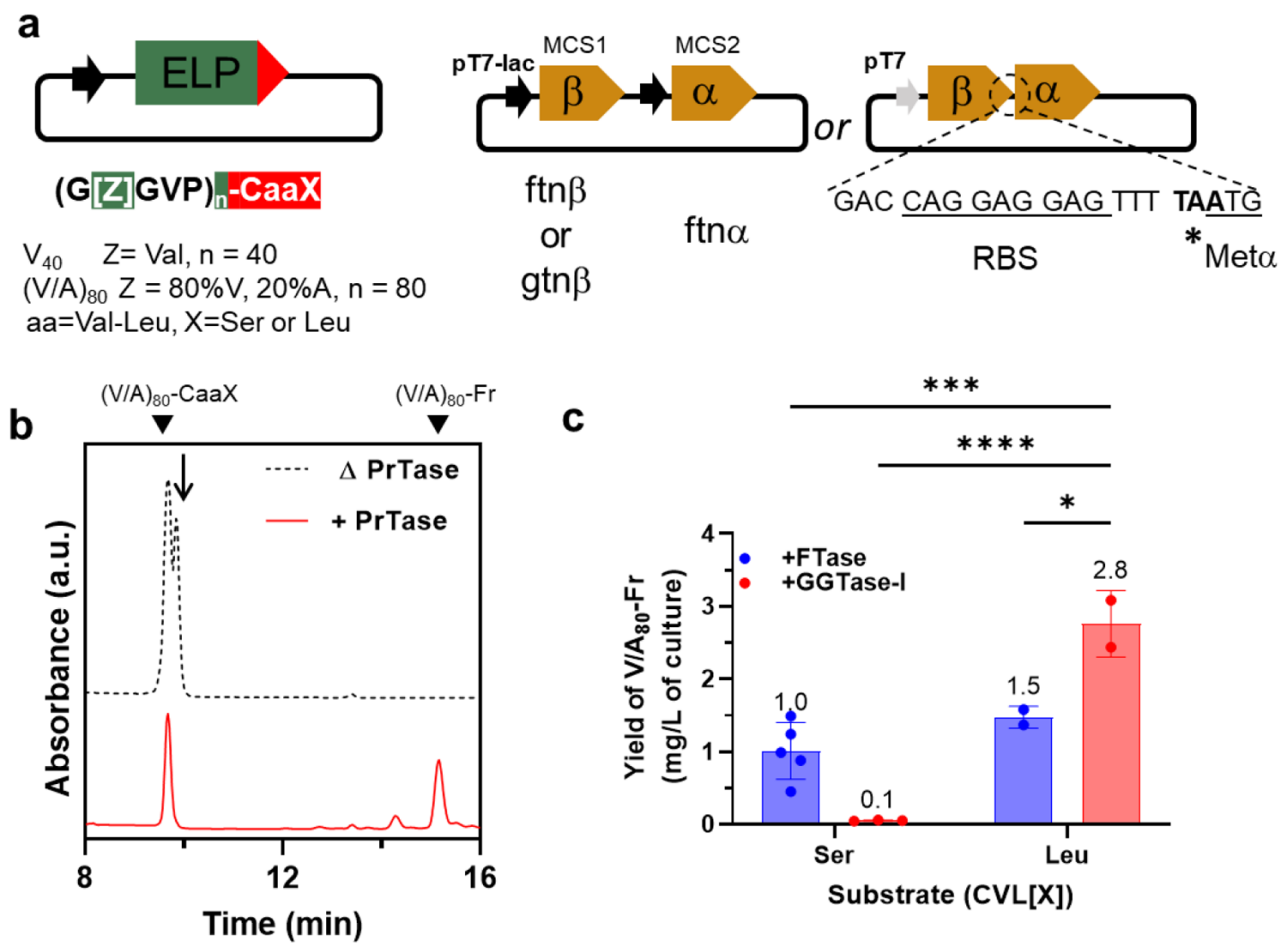
(a) Architecture of plasmids used for the biosynthesis of farnesyl-modified ELPs. Two compatible plasmids were used to encode all genetic elements necessary for in vivo farnesylation: ELPs fused to two canonical CaaX motifs, CVLS and CVLL; α and β subunits for FTase or GGTase-I, which together constitute the heterodimeric prenyltransferase. These subunits were expressed independently (from the bicistronic pETDuet-1 vector) or translationally coupled by including ribosomal binding site and start codon (underlined) for the α-subunit at the end of the β-subunit. (b) Representative RP-HPLC chromatogram for (V/A)_80_ (fused to CVLL) expressed in the absence (dashed black line) or the presence (solid red line) of prenyltransferase. The peak marked with an arrow corresponds to the dimer of (V/A)_80_, formed via a disulfide bond between the cysteine residue in CaaX motif. (c) Comparison between the cumulative yields of farnesylated protein produced from coexpression of FTase *or* GGTase-I with (V/A)_80_-CVLX (X = Ser or Leu). While FTase can farnesylate (V/A)_80_ fused to either substrate, GGTase-I can produce the highest yield of (V/A)_80_-Fr from substrate bearing CVLL peptide, 2–3× the amount produced by the canonical enzyme (FTase). Error bars in c represent standard deviations of 2–5 independent replicates. One-way ANOVA with post hoc Dunnett’s test for multiple comparisons HSD test; *, ***, and **** signify *p* < 0.05, .001, and .0001.

We employed an iterative “design, build, and test” process to reconstitute and optimize the protein-farnesylation platforms. For this proof-of-concept demonstration, we used endogenously produced FPP as the lipid donor to minimize the number of expressed proteins to three: model protein (i.e., ELP) fused to CaaX sequence, and the alpha and beta subunits of FTase or GGTase-I. FTase and GGTase-I are heterodimeric proteins with identical α but divergent β subunits. FPP is the canonical lipid donor for FTase, but it is also accepted by GGTase-I.^46^ We selected the two prenyl transferase subunits from *R. norvegicus* as they have been previously expressed in *E. coli*.^47^ Genes encoding α and β subunits as well as model ELP fused to short peptide sequences (CVLS and CVLL) were cloned into a set of orthogonal plasmids with compatible origins of replication (pBR322 and p15A) and selection markers (Kan^r^ and Amp^r^) using Gibson assembly and recursive directional ligation (Figure 2a). Two ELPs with different lengths and hydrophobicity were chosen for this study. The first ELP contains 40 repeats of GVGVP units (hence referred to as V_40_), while the other ELP is comprised of 80 repeats of GZGVP with the composition of *Z* = 80% V and 20% A, referred to as (V/A)_80_. This ELP also contained eight lysine residues distributed throughout the sequence.

To identify conditions for high-yield heterologous production of the transferase, we initially used the pETDuet-1 vector to coexpress the prenyltransferase subunits in BL21(DE3) strains. This bicistronic vector uses two T7/lac promotors to *independently* produce α and β subunits. Despite the high expression efficiency of this vector, almost all the expressed transferase was found in insoluble inclusion bodies (Figure S1). Unfortunately, changing the expression media, temperature, inducer concentration, and induction time did not increase the soluble protein production (data not shown). Since the individual subunits of prenyltransferase tend to aggregate when not bound to their complementary subunits,^48^ we next used a translational coupling system to link the production of α and β subunits. In this construct, the stop codon (TAA) of the β-subunit overlaps with the start codon of α-subunit, i.e., *TA*ATG. This translationally coupled system had previously been used to produce the F/GG-Tase in *E. coli* at quantities sufficient for biochemical characterization.^49,50^ We verified that this plasmid produces recombinant, soluble prenyl transferase in *E. coli*, which can lipidate its protein substrate in vitro (Figure S2).

The laborious and costly synthesis of FPP hinders the in vitro production of farnesylated proteins at a scale sufficient for various biomedical and materials applications. Therefore, we investigated whether coexpression of protein substrate and the transferase can be used to recombinantly produce desired farnesylated proteins in a one-pot expression-lipidation system (Figure 2b). We used HPLC to determine the ratio of lipidated and nonlipidated ELP-CaaX in the absence or presence of prenyltransferase. When ELP-CaaX is expressed alone, the protein elutes at 9.7 min (the peak at 9.8 min, marked with an arrow, corresponds to disulfide-bonded dimer). When ELP-CaaX is coexpressed with prenyltransferases, the intensity of the unmodified peak was reduced, and another peak with a longer retention time (15.2 min) was observed on the chromatogram. The increased retention time is consistent with the increased hydrophobicity of the lipidated protein. Additional molecular characterization (*vide infra* for details) was consistent with the assignment of this peak to the farnesylated product.

Having established that one-pot recombinant expression of farnesyl-modified proteins is possible, we then focused on maximizing the yields of recombinant farnesylated proteins. Several intrinsic factors influence the production yield, including (i) the concentration of the intracellular pool of FPP; (ii) the binding specificity of FTase and GGTase-I toward FPP (*K*_m_); and (iii) the rate of lipid transfer (*k*_cat_) to the protein of interest, which depends on the identity and “X”-residue in the CaaX motif.^51^ Therefore, we first used a 2 × 2 factorial design to quantify how enzyme/substrate combinations—[FTase or GGTase-I]/[X = Ser or Leu]—influence the production yield of (V/A)_80_-Fr, in mg/L of culture (Figure 2c). In this platform, FTase modified proteins fused to both CVLS and CVLL (its canonical and noncanonical substrates) with similar efficiency. On the other hand, GGTase-I only modified (V/A)_80_-CVLL and not (V/A)_80_-CVLS. One-way ANOVA revealed a statistically significant difference between at least two enzyme/substrate combinations (*F*(3, 8) = 28.73, *p* = 0.0001). While FPP is not the canonical lipid donor for GGTase-I, a post hoc Dunnett’s Test for multiple comparisons revealed that the GGTase-I/CVLL combination had the highest yield compared to other combinations of enzyme/substrate, i.e., GGTase-I/CVLS (*p* < 0.0001, 95% C.I. of differences = [1.9–3.6]; FTase/CVLS (*p* = 0.0005, 95% C.I. = [1.0–2.5]), and FTase/CVLL (*p* = 0.01, 95% C.I. = [0.4–2.2]). Moreover, the farnesylation efficiency (i.e., the percentage of lipidated ELP) was inversely correlated with the protein size (Figure S3), 37% for (V/A)_80_, and 86% for V_40_. We attribute this observation to the reduced accessibility of the CaaX motif when it is fused to larger proteins.^52^

Based on these experiments, we proceeded with GGTase-I/CVLL for scaled-up production of farnesylated proteins (V_40_-Fr and (V/A)_80_-Fr) and their nonlipidated controls in a conventional biosynthetic platform. Briefly, plasmids encoding the α and β subunits of GGTase-I and substrate protein fused to CVLL were cotransformed to BL21(DE3) *E. coli* strains. The bacteria were cultivated at 37 °C to OD_600_ ∼ 0.8 before reducing the temperature to 28 °C. At this point, the expression culture was supplemented with IPTG (1 mM, to induce the coexpression of proteins) and ZnSO_4_ (0.5 mM, metal cofactor required for GGTase-I). After 18 h, cells were harvested by centrifugation. We isolated the post-translationally lipidated ELPs by adapting a method developed by Thompson and co-workers.^45^ This approach uses a combination of organic (non)solvents to lyse the cells and selectively isolate the ELP from the complex mixture of cell lysate proteins. Our work here shows that post-translationally lipidated ELPs can also be purified using this method.

**Figure 3:**
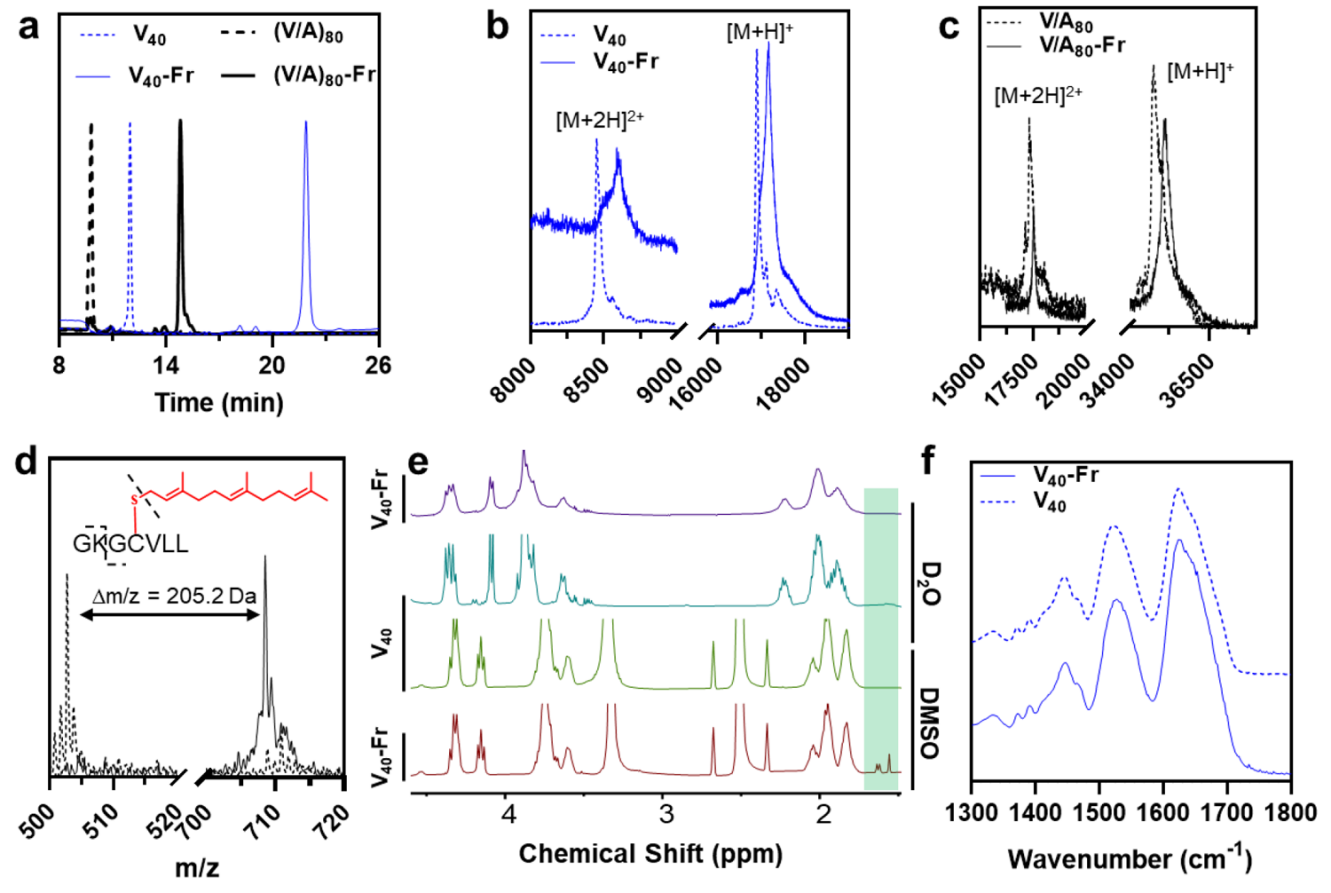
(a) Reverse-phase HPLC chromatograms of unmodified and farnesylated V_40_ and (V/A)_80_ confirm the purity of each construct and increased hydrophobicity of farnesylated proteins. (b,c) The MALDI-TOF-MS analysis is consistent with the addition of a single farnesyl group to each protein. (d) The location of the farnesyl group is confirmed by the digestion of the (V/A)_80_-Fr with trypsin and the analysis of peptide fragments using MALDI-TOF-MS. The molecular weight of the C-terminus peptide fragment (GCVLL) is increased by 205.2 Da, corresponding to the mass of the Fr group. (e) ^1^H NMR spectra of V_40_ and V_40_-Fr in D_2_O and DMSO-*d*_6_. The broadening of V_40_-Fr peaks in D_2_O (purple line) is due to the lipid-induced oligomerization of V_40_-Fr. The green band highlights the position of allylic methyl groups in the farnesyl group. (f) Attenuated total reflectance FT-IR spectra of the amide (I–III) region of V_40_ and V_40_-Fr. The broad bands in this region are consistent with the presence of large fractions of disordered domains in the lyophilized state of both proteins, showing that farnesylation does not alter the structure of ELP at the chain level.

After purification, we used a combination of HPLC, MALDI-TOF MS, and NMR to verify constructs’ identities and purities and to establish that only one farnesyl group is added to the protein at the cysteine residue located in the C-terminal CaaX motif (Figure 3). As shown in Figure 3a, analytical RP-HPLC confirms the purity of each construct and provides a measure of their hydrophobicity based on their retention time. As expected, farnesylated proteins had longer retention times than their unmodified analogs, consistent with their increased hydrophobicity. The unmodified V_40_-CVLL eluted at 12.0 min (Figure 3a, dashed blue curve), while the V_40_-Fr eluted at 21.9 min (solid blue curve). The unmodified (V/A)_80_-CVLL eluted at 9.8 min (dashed black curve), while (V/A)_80_-Fr eluted at 14.8 min (solid black curve).

For both ELPs, the molecular ion peak corresponding to lipidated protein was shifted by *m*/*z* = +205.2 Da, consistent with adding a farnesyl motif (and removing a hydrogen atom from the thiol). In each case, we also detected the doubly charged ion [M + 2H]^2+^ (Figure 3b,c). To identify the location of the modification, (V/A)_80_-Fr was digested with trypsin, and the resulting peptide fragments were analyzed with MALDI-TOF-MS. Trypsin cleaves the peptide backbone after positively charged amino acids, such as lysine. There are eight lysines in (V/A)_80_, which are distributed throughout the sequence of ELPs, with one located before the CVLL recognition sequence. As shown in Figure 3d, we observed a peak at *m*/*z* = 708.5 Da, which was assigned to *S*-farnesylated GCVLL. This peak was not present in the nonlipidated control, which instead contained a peak at *m*/*z* of 502.8 Da, corresponding to the unmodified peptide.

Because V_40_-Fr lacked a trypsin digestion site near the farnesylation site, we used ^1^H NMR spectroscopy to confirm its farnesylation. The spectra of V_40_ and V_40_-Fr in D_2_O are shown in Figure 3e. Even though the sequence of ELPs is highly repetitive, their sequence-defined and monodispersed nature often results in sharp NMR peaks (cyan spectra). However, the spectra of V_40_-Fr in D_2_O exhibited broad peaks (purple spectra) and lacked signals corresponding to the farnesyl group. We hypothesized that the broad peaks are due to the lipid-induced oligomerization of the proteins,^53^ which reduces molecular tumbling and both longitudinal and transverse relaxation rates of micellar assemblies. We, therefore, collected the spectra of V_40_ and V_40_-Fr in deuterated DMSO as this organic solvent can disrupt the hydrophobic core of assemblies, resulting in the formation of freely diffusing protein chains (i.e., soluble unimers). As shown in Figure 3e, spectra collected in DMSO confirm this hypothesis as both V_40_ and V_40_-Fr gave rise to sharp peaks (green and maroon spectra). Moreover, signals corresponding to the farnesyl’s allylic methyl groups were clearly visible in 1.5–1.7 ppm, and their integration matched the theoretical one farnesyl group per protein chain (Figure S4).

Finally, we used FT-IR to see if lipidation perturbs the structure of ELPs by comparing the amide absorption bands of unmodified and farnesylated V_40_ (Figure 3f). In each case, the FT-IR absorption band maxima were consistent with the presence of large, disordered protein domains, consistent with the absence of significant secondary structure after lipidation. We were unable to detect the weak absorption of farnesyl C=C or C–H stretches in the FT-IR, which were likely buried by the strong signals originating from the polypeptide structure.

**Figure 4:**
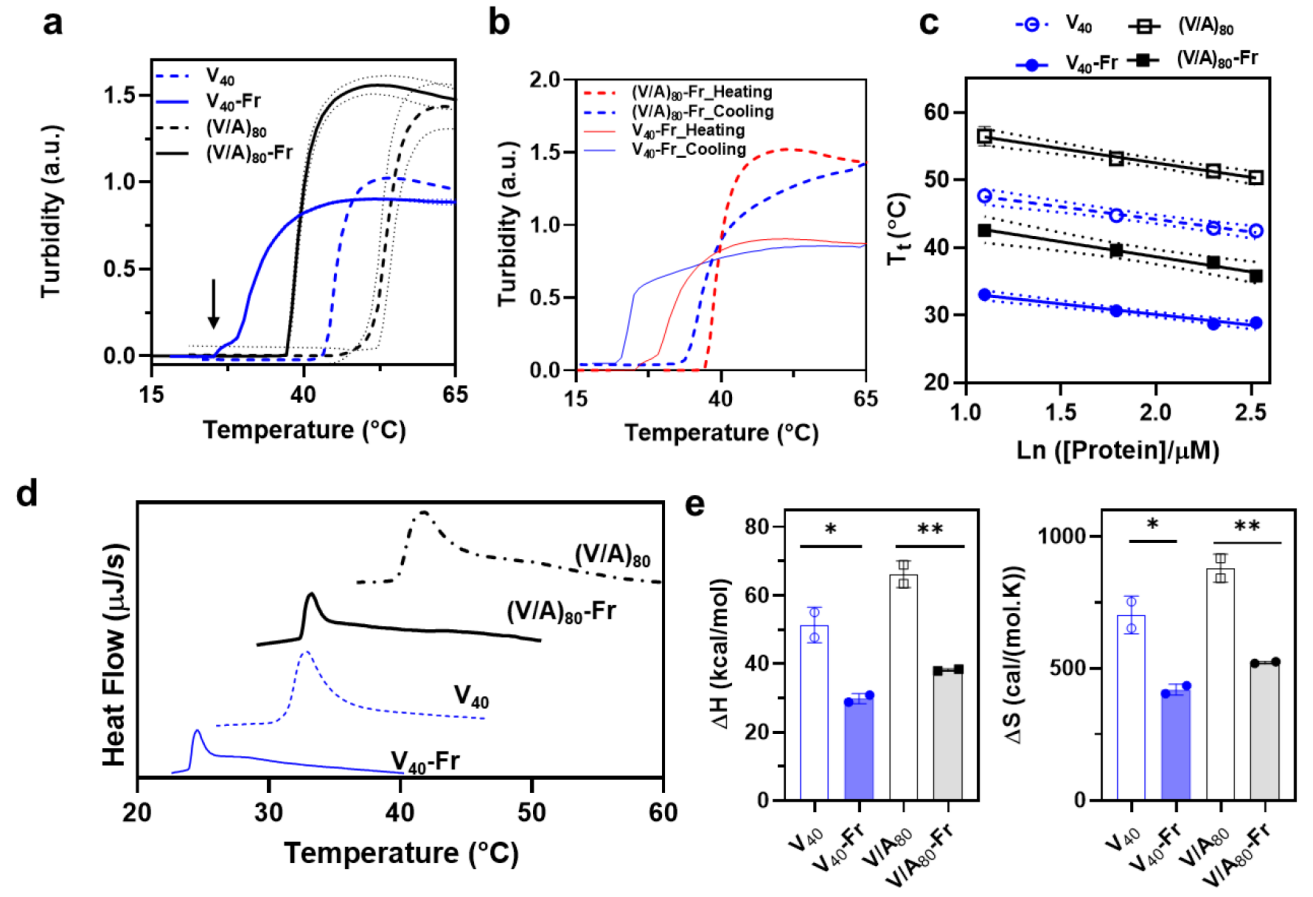
Characterization of the effect of farnesylation on ELP’s thermoresponse in PBS using turbidimetry and differential scanning calorimetry. (a) Turbidity profiles of unmodified (dashed) and farnesylated (solid) V_40_ and (V/A)_80_, blue and black traces, respectively. Dotted lines represent the standard deviations of two independent measurements. (b) Temperature-programmed turbidimetry is used to monitor the reversibility of the LCST phase-transition of V_40_-Fr (solid) and (V/A)_80_-Fr (dashed) after one cycle of heating and cooling (red and blue curves). Both lipidated proteins exhibited reversible temperature-triggered phase behavior. [protein] = 6 μM in a and b. (c) Concentration dependence of the proteins’ transition temperatures. The *T*_t_ vs natural log of protein concentration is fitted using a linear regression model. Dotted lines represent a 95% confidence interval for the fitted line. (d) DSC thermograms of the unmodified (dashed) and farnesylated (solid) proteins, V_40_ (blue) and (V/A)_80_ (black). The endothermic peak corresponds to the temperature-triggered phase separation of each construct. Farnesylation reduces the AUC but increases the asymmetry of the peak. (e) Thermodynamic parameters (enthalpy and entropy of the phase separation) for each construct plotted on a bar graph. Farnesylation reduces the enthalpy and entropy of phase separation of ELPs (two-tailed unpaired *t* test, * *p* < 0.05, ** *p* < 0.01). The error bars are standard deviations of two independent measurements.

Biopolymers with programmable thermoresponse (such as ELPs) are attractive materials for biomedical applications because the temperature can be increased locally as a therapeutic modality while causing minimal damage to healthy tissues.^54^ While the effect of amino acid mutations on the liquid–liquid phase separation of proteins is under intense investigation,^55,56^ our understanding of the effect of lipidation on the phase-boundaries remains incomplete. Given the importance of thermoresponse in biomedical applications,^57,58^ we used complementary techniques of turbidimetry and DSC to quantify the effect of farnesylation on the temperature-triggered phase separation of elastin-based proteins. Figure 4a shows the turbidity of the solution of nonlipidated and farnesylated ELPs (6 μM in PBS) as a function of temperature. All proteins, except for V_40_-Fr, show a rapid and one-step transition, characterized by the rapid increase in solution turbidity as the temperature increases above the LCST transition temperature (*T*_t_). The turbidity of the solution of V_40_-Fr is initially increased modestly around 27 °C (marked with an arrow in Figure 4a, solid blue curve), followed by a sharp increase when *T* > 29 °C. We note that the increased turbidity of (V/A)_80_, in comparison to V_40_, is due to its larger molecular weight and protein weight fraction,

Turbidimetry was also used to investigate the reversibility of the phase separation of farnesyl-modified proteins (Figure 4b) by comparing the evolution of turbidity profiles during heating and cooling cycles (red and blue curves). Similar to their nonlipidated analogs (Figure S5), the phase separation of V_40_-Fr and (V/A)_80_-Fr was completely reversible as the turbidity of the solution reached its initial values before heating, albeit V_40_-Fr exhibited slight hysteresis as evident by the area between the dashed heating and cooling curves. This kinetic effect is likely due to the combination of farnesyl-mediated oligomerization and increased hydrophobicity of V_40_ (compared to (V/A)_80_), which strengthens ELP–ELP interactions after coacervation and increases the barrier for dissolution of coacervates.

We also evaluated the effect of farnesylation on the concentration dependence of *T*_t_ for both constructs. Quantifying this behavior is critical for developing models that can reliably predict the behavior of farnesylated constructs in biomedical applications in which the concentration of protein changes as a function of time (e.g., thermally triggered drug depots, intravenous administrations, or hyperthermia-based targeting and treatment of cancer).^59−61^ Turbidimetry was used to measure the transition temperature for unmodified and farnesylated proteins at various concentrations (3, 6, 10, 12.5 μM in PBS), Figure S6. Transition temperature was defined as the inflection point (i.e., the maximum of the first derivative, dAbs/dT) and was plotted against the natural log of protein concentration to develop a temperature–composition portrait (Figure 4c). For all constructs within this concentration range, the transition temperature changed linearly with the natural log of the protein concentration (Table S3). Farnesylation did not change the concentration dependence of T_t_ (i.e., the slope of the line) as no statistically significant difference between slopes of the nonlipidated and farnesylated proteins were observed (ANCOVA for V_40_ constructs: *F*(1, 8) = 1.37 *p* = 0.3; for (V/A)_80_ constructs: *F*(1, 8) = 0.07, *p* = 0.8). On the other hand, farnesylation reduced the *Y*-intercept (i.e., *T*_t_) by 15.2 °C for V_40_ and 13.8 °C for (V/A)_80_, a statistically significant difference between nonlipidated and farnesyl-modified proteins, ANCOVA for V_40_ constructs: *F*(1, 9) = 2705, *p* < 0.0001; for (V/A)_80_ constructs *F*(1, 9) = 1107, *p* < 0.0001.

To investigate how farnesylation modulates the thermodynamics of liquid–liquid phase separation, we used DSC to measure the total heat of phase separation. Figure 4d presents the DSC thermograms of nonlipidated and farnesyl-modified V_40_ and (V/A)_80_ constructs. An endothermic peak distinguishes the LLPS of ELP, and the area under this peak corresponds to the Δ*H* of the phase separation process. Farnesylation reduces the peak’s area and notably increases its asymmetry. This observation suggests that farnesylation not only changes the thermodynamics of phase separation but may also change processes such as nucleation, coalescence, or ripening of coacervates. Given the positive Δ*H* of phase separation for both lipidated and nonlipidated proteins, the driving force for phase separation is the favorable and positive entropy (Δ*S*) for releasing “frozen” water molecules in the hydration shell of proteins. DSC also revealed that farnesylation lowers the enthalpy (Δ*H*) of coacervation for both constructs (two-tailed unpaired *t* test; (V_40_ vs V_40_-Fr): *t*(2) = 5.63, *p* = 0.03; (V/A)_80_ vs (V/A)_80_-Fr: *t*(2) = 10.09, *p* = 0.0097) and thus have a stabilizing effect (−22 to −28 kcal/mol) on the coacervates (Figure 4e, Table S4). This is likely through favorable van der Waals interactions between farnesyl groups (i.e., the Δ*H* of micellization < 0) or because it changes the interactions of ELP chains within the micelle.^62^

**Figure 5:**
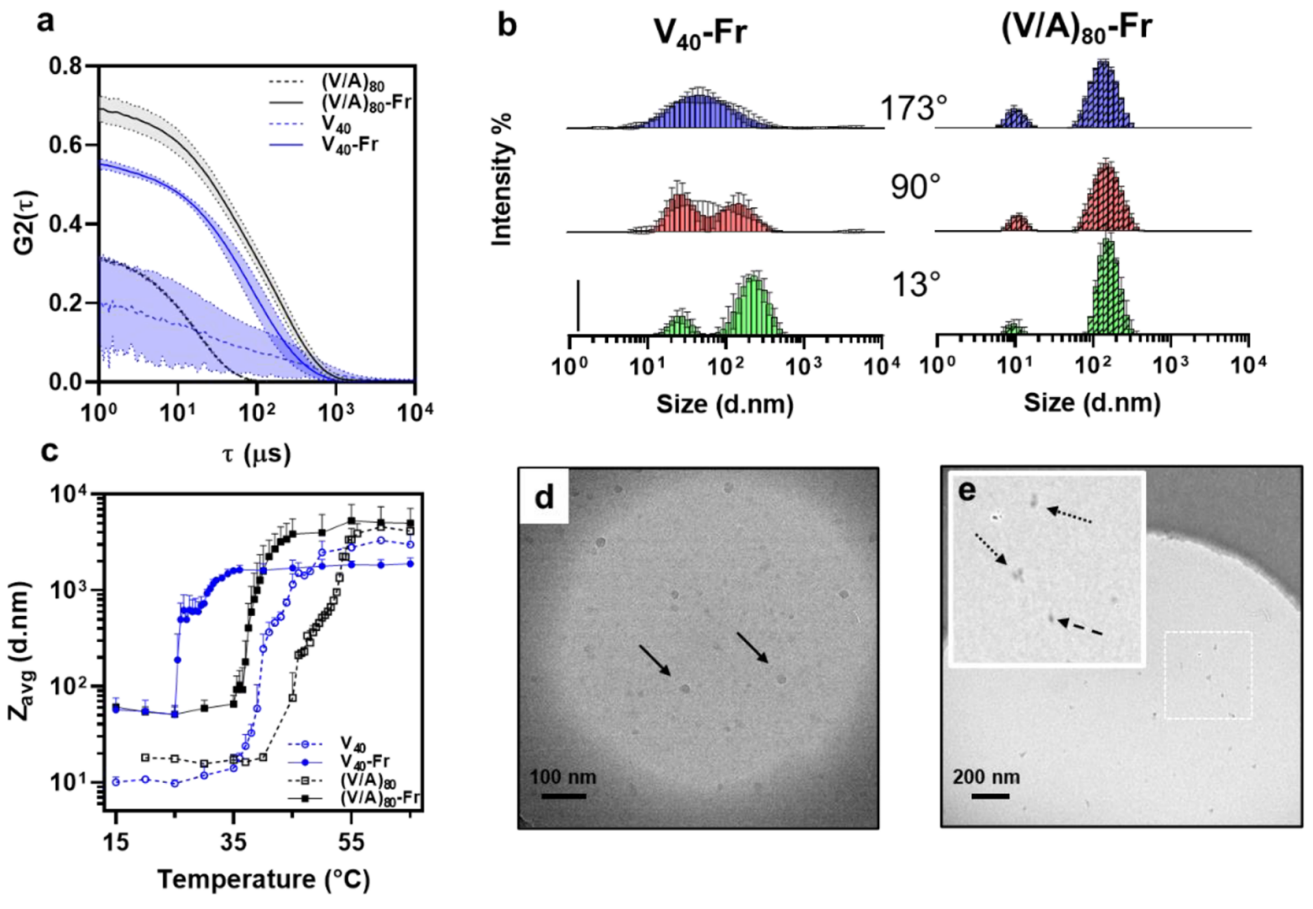
Characterization of nanoassembly of farnesylated proteins in PBS using dynamic light scattering and cryo-TEM. (a) Autocorrelation functions of unmodified (dashed) and farnesylated (solid) constructs at 20 °C (blue V_40_, black (V/A)_80_). The shift in ACFs of unmodified and lipidated proteins is consistent with the changes in the oligomerization of proteins after farnesylation. (b) The intensity-size distribution of V_40_-Fr and (V/A)_80_-Fr at 20 °C derived from the analysis of scattering fluctuations collected at 13°, 90°, and 173° using CONTIN algorithm. The vertical scale bar corresponds to 10%. (c) DLS is used to monitor the changes in the average hydrodynamic size of constructs as a function of temperature. The unmodified proteins size corresponds to the size of unassembled unimers below *T*_t_, while all lipidated samples formed larger assemblies. Above *T*_t_, both unmodified and farnesylated proteins formed mesoscale coacervates. [protein] = 6 μM in a–c. Cryo-TEM images of V_40_-Fr (d) and (V/A)_80_-Fr (e) dissolved in PBS at 100 μM. V_40_-Fr formed spherical micelles (diameter = 13.3 ± 3.0 nm) whereas (V/A)_80_-Fr formed smaller micelles (diameter 9.1 ± 2.7 nm), which further assembled to form supra-particles. The error bands (a) and bars (c) represent the standard deviation of six measurements (two independent samples, each measured in triplicate). The error bars in b are standard deviations of three measurements at each angle.

As discussed earlier, the broad peaks in the NMR (Figure 3e) and reduced enthalpy and entropy of phase separation (Figure 4e) suggest that farnesylated constructs may self-assemble. Therefore, we investigated the effect of farnesylation on the nanoscale assembly of these constructs using multiangle dynamic light scattering (MADLS) and cryogenic transmission electron microscopy (cryo-TEM). Figure 5a shows the autocorrelation functions (ACFs) derived from analysis of raw scattering data collected at 173°. The ACFs of unmodified ELPs are characterized by a fast decay and low *Y*-intercept (<0.5). On the other hand, the ACFs of farnesylated proteins exhibited a slower decay (increase in the *X*-intercept) and higher *Y*-intercept (>0.5), consistent with the formation of larger assemblies that scatter light more strongly.

The visual inspection of ACFs for farnesyl-modified proteins also shows that the exponential decay profile is not monotonic, which likely is due to equilibrium between assemblies of different sizes. The bimodal size-intensity distributions at different angles (13°, 90°, and 173°) shown in Figure 5b are consistent with this conjecture. Because the larger particles scatter light more strongly at smaller angles, the MADLS profile can be used to improve the resolution between the two populations (c.f., V_40_-Fr distributions at 173° vs 13°). This dynamic equilibrium is distinct from the behavior of supramolecular assemblies formed by proteins modified with fatty acids or sterols, which formed static assemblies with shapes and sizes governed by their molecular composition.^43^

To determine the effect of temperature on the properties of these assemblies, DLS was conducted from 15–65 °C at 5 °C increments. The ACF at each temperature was analyzed using the cumulant method to calculate the average hydrodynamic diameter of proteins (Figure 5c, Table S5). In each case, the hydrodynamic radius increased by 1 to 2 orders of magnitude *T* > *T*_t_, consistent with the formation of mesoscale coacervates at higher temperatures (Figure S7). We note the behavior of V_40_-Fr constructs was slightly different; the temperature-triggered increase in the hydrodynamic radius appeared to occur within two distinct stages with the first increase at 25–28 °C followed by a secondary increase at *T* > 28 °C, a behavior consistent with the turbidimetry results. Moreover, DLS also confirmed that the temperature-triggered phase-separation of both unmodified and lipidated proteins is reversible (Figure S8).

Cryo-TEM was used to image the assemblies of farnesylated proteins at 20 °C (below their transition temperature). Intriguingly, we observed noticeable differences in the nanostructure of V_40_-Fr and (V/A)_80_-Fr, even though DLS yielded similar *Z*_avg_ for both constructs. As shown in Figure 5d (see Figure S9 for the histogram depicting the size distributions), V_40_-Fr formed spherical nanoparticles with an average size of 13.3 ± 3.0 nm (Figure 5d, solid arrows), which exhibited relatively uniform contrast (i.e., hydration level). On the other hand, the cores of (V/A)_80_ assemblies were smaller, 9.1 ± 2.7 nm (Figure 5e, dashed arrow), but we observed supra-particles that appeared to form from the association of these smaller particles (Figure 5e, dotted arrows). We note that DLS and TEM provide complementary information about the nanoscale assembly of farnesylated ELPs.^63^ DLS characterizes the average hydrodynamic diameter and the size-distributions of Fr-modified nanoparticles. Cryo-TEM, however, visualizes assemblies based on differences in their hydration. Since ELP chains are hydrated below their transition temperature, cryo-TEM may only visualize the hydrophobic core of the assemblies, resulting in a smaller apparent diameter. On the other hand, the most common algorithms for the analysis of DLS data (cumulants and CONTIN) assume that the assemblies are spherical and are not able to estimate the size of more complex assemblies (e.g., supraparticles of (V/A)_80_-Fr) accurately.

Finally, we characterized the mesoscale assembly of farnesyl-modified proteins above their *T*_t_ using differential interference contrast (DIC) microscopy. Like their unmodified analogs, farnesylated proteins underwent liquid–liquid phase separation to form protein-rich coacervates (droplets), albeit farnesylation altered the average size (size-distribution) of coacervates (Figure S10).

## Conclusion

By converging synthetic biology with materials science, we developed recombinant platforms to produce farnesylated proteins with programmable assembly and temperature-dependent characteristics. Although in vitro and lysate prenylation of proteins has been previously demonstrated, the laborious and expensive synthesis of prenyl donors limits the scalability of these methods. Consequently, they are typically used to produce small quantities (a few μg) of naturally occurring farnesylated proteins for biochemical characterization or chemoenzymatic labeling of proteins with bio-orthogonal handles. Our design–build–test approach enables scalable production of farnesylated proteins outside of the biological context (i.e., artificial proteins and noncanonical lipidation sites) for biomaterial and biomedical applications. FPP is not the preferred canonical substrate of GGTase-I;^64,65^ however, our results show that the combination of GGTase-I/CVLL can efficiently produce farnesylated proteins in *E. coli*. This observation likely reflects the differences in the concentration and availability of prenyl donors inside a microbial factory from the conditions used to characterize the biochemistry of enzymes in vitro (e.g., excess lipid donor or peptide substrate).

By combining biophysical and soft-matter characterization techniques, we show that farnesylation alters the thermoresponse, phase separation, and assembly of ELPs used as model systems. Dynamic light scattering and cryo-TEM show that the farnesylated ELP assemblies exist in a dynamic equilibrium between unimers and assembled structures, a behavior distinct from the assemblies of ELPs modified with other lipids, such as saturated fatty acids^66^ or sterols.^42^ We propose that increased dynamics of ELP-Fr micellar assemblies is due to the reduced hydrophobicity and packing efficiency of the unsaturated farnesyl group. This observation suggests that the physicochemical properties of the lipid can be used as a design principle to control the biophysical and material properties of this class of hybrid materials.

In principle, our methodology can be used to produce farnesylated proteins from both globular proteins (e.g., enzyme and nanobodies) as well as natural and artificially engineered protein polymers^67^ (e.g., resilin,^68^ silk,^69,70^ abductin,^71^ among others) using a conventional biosynthetic platform. Lipid modification of high molecular weight biopolymers has remained largely unexplored in the field of materials science because current synthetic methods are technically challenging, time-consuming, and expensive.^72^ Our biosynthetic platform addresses these limitations, paving the road to developing the next generation of hybrid functional biomaterials whose forms and functions rival the exquisite hierarchy and capabilities of biological systems.

To make this technology scalable (and commercially competitive), it is necessary to increase the yield (>10 mg/L of culture) and farnesylation efficiency (>90%) by metabolic and genetic engineering of production strains. More work is also needed to develop a generalizable method for isolating farnesylated proteins from endogenous lipid membranes and unmodified products. In this paper, we leveraged the organic extraction and liquid–liquid phase separation for scalable and nonchromatographic purification of farnesylated products. Proteins without phase behavior can be purified by membrane fractionations^73^ or partitioning into surfactant-rich phases^74^ to preserve their native structure and activity.

Protein farnesylation remains an untapped resource in our synthetic and therapeutic toolkit.^75^ The development of operationally simple, high-yield biosynthetic routes to produce farnesylated proteins and reveal biophysical determinants of their assembly opens the path to developing recombinant biomaterials and therapeutics. For instance, farnesylated proteins are genetically encoded amphiphiles that can form nanoparticles with tunable characteristics for encapsulation of hydrophobic therapeutics (e.g., paclitaxel) or complexation with nucleic acids (e.g., small interfering RNA). The sequence of these biodegradable nanobiomaterials can be controlled with precision to modulate carriers’ characteristics and in vivo targeting. Finally, because farnesylation exclusively occurs at the c-terminus of proteins, these recombinant nanoparticles can be used to display biologically active peptides that interact with their cognate receptors with their N-terminus (e.g., exendin, calcitonin, secretin, and other peptide ligands for class B G-protein-coupled receptors).^76^

Recent studies have greatly expanded the size of potential prenylated proteome beyond the classic CaaX sequence to other motifs such as C(x)_3_X and Cxx.^77,78^ Nonetheless, the correlation between the sequence of these lipidation sites and the assembly or localization of prenylated proteins remains less clear. This paper sets the stage for future studies to reveal the interplay of prenylation sites or the effect of postprenylation processing steps^79^ on the emergent properties of farnesylated proteins. Moreover, our strategy can be applied to the biosynthesis of geranylgeranylated proteins by metabolically engineering *E. coli* to increase the intracellular GGPP. These studies are underway in our laboratories and will be reported in due course.

## Supporting Information Available

Materials, sequences of proteins, detailed experimental protocols, supporting tables, and supporting figures (PDF)

## Abbreviations

ACF: autocorrelation function
DLS: dynamic light scattering
ELP: elastin-like polypeptides
LCST: lower critical solubility temperature
PDI: polydispersity index
PTM: post-translational modifications
PBS: phosphate buffered saline
TEM: transmission electron microscopy
