# Supplementary material for: Temperature-Responsive Nano-Biomaterials from Genetically Encoded Farnesylated Disordered Proteins
Source: ACS Appl Bio Mater. 2022 Jan 19;5(5):1846–56. doi: 10.1021/acsabm.1c01162 (PMC9115796; doi:10.1021/acsabm.1c01162)
Supplement: Supplementary file 1 — mt1c01162_si_001.pdf [file mt1c01162_si_001.pdf]

Supporting Information

Temperature-Responsive Nano-Biomaterials from Genetically Encoded Farnesylated Disordered Proteins

Md. Shahadat Hossain[1], Zhe Zhang[1], Sudhat Ashok[1,‡], Ashley R. Jenks[1], Christopher J. Lynch[1], James L. Hougland[1,2,4], and Davoud Mozhdehi[1,2,3,4*]

1. Department of Chemistry, Syracuse University, Syracuse, New York 13244, United States; 2. Department of Biology, Syracuse University, Syracuse, New York 13244, United States; 3. Department of Biomedical and Chemical Engineering, Syracuse University, Syracuse, New York 13244, United States; 4. BioInspired Syracuse: Institute for Material and Living Systems, Syracuse University, Syracuse, New York 13244, United States; ‡ Current position: Jacobs School of Medicine and Biomedical Sciences, University of Buffalo, Buffalo, New York 14203, United States.

E-mail: dmozhdeh@syr.edu

Contents

1. Materials 2
2. Cloning 2
3. Protein sequence 3
4. Protein purification 4
5. Molecular Characterization 4
   5.1 Analytical HPLC 4
   5.2 Matrix-Assisted Laser Desorption/Ionization Time-of-Flight Mass Spectrometry (MALDI-TOF-MS) 5
   5.3 Trypsin Digestion 5
6. Turbidimetry assay 5
7. Supplementary Tables 6
8. Supplementary Figures 8
9. References 15

1. Materials

The pACYCDuet-I vector was purchased from EMD Millipore (Billerica, MA). The chemically competent Eb5alpha and BL21(DE3) cells, restriction enzymes, ligase, and corresponding buffers, as well as DNA extraction and purification kits, were purchased from New England Biolabs (Ipswich, MA). Isopropyl β-D-1-thiogalactopyranoside (IPTG) was purchased from A. G. Scientific (San Diego, CA). Apomyoglobin, adrenocorticotropic hormone (ACTH), sinapinic acid, alpha-cyano-4-hydroxycinnamic acid, zinc sulfate, and trifluoroacetic acid (TFA) were purchased from Sigma-Aldrich (St. Louis, MO). High-performance liquid chromatography-(HPLC) grade acetonitrile, SnakeSkin™ dialysis tubing with 3.5K nominal molecular weight cut off (MWCO), mass spectroscopy grade Pierce™ trypsin protease, tryptone, yeast extract, sodium chloride, ampicillin, kanamycin, chloramphenicol, phosphate buffer saline (PBS), DMSO, isopropanol, acetonitrile, and ethanol were purchased from Thermo Fisher Scientific (Rockford, IL). Mini-PROTEAN® TGX Stain-Free™ Precast Gels, Precision Plus Protein™ All Blue Pre-stained Protein Standard, and Precision Plus Protein™ Unstained Protein Standards were purchased from Bio-Rad Laboratories, Inc. (Hercules, CA). Deionized water was obtained from a Milli-Q® system (Millipore SAS, France). Simply Blue™ SafeStain was purchased from Novex (Van Allen Way Carlsbad, CA). All chemicals were used as received without further purification.

2. Cloning

The sense and antisense oligonucleotides encoding for the canonical peptide substrate of FTase (CVLS) and GGTase-I (CVLS) were purchased from IDT. The 5′-ends of single-stranded DNAs were phosphorylated, after which the complementary oligos were thermally annealed. The double-stranded DNA was then cloned into a modified pET24a(+) vector, referred to as pJMD5, which had been double digested with BseRI and BamHI. Recursive directional ligation by plasmid reconstruction was used to fuse the peptide substrates to the C-termini of V_{40} and $(V/A)_{80}$ genes.[1] After sequence verification, the ELP-CVLS or ELP-CVLL were cloned into pACYCDuet-I using NdeI and XhoI restriction enzymes. Genes encoding for α and β subunits of FTase-I and GGTase-I were ordered as gene fragments from IDT DNA and were cloned into pETDuet-1 using Gibson assembly. The beta subunit was cloned into MCS1 between NcoI and EcoRI sites, and the alpha subunit was cloned into MCS2 between Nde and XhoI. The construction of vectors used for translational coupling of α and β subunits has been reported previously.[2, 3]

3. Protein sequence

V$_{40}$

*(M)*GVGVPGVGVPGVGVPGVGVPGVGVPGVGVPGVGVPGVGVPGVGVPGVGVPGVGVPGVGVPGVG
VPGVGVPGVGVPGVGVPGVGVPGVGVPGVGVPGVGVPGVGVPGVGVPGVGVPGVGVPGVGVPGVG
VPGVGVPGVGVPGVGVPGVGVPGVGVPGVGVPGVGVPGVGVPGVGVPGVGVPGVGVPGVGVPGVG
VPGVGVPGCVLL

(V/A)$_{80}$

*(M)*GVGVPGVGVPGAGVPGVGVPGVGVPGVGVPGVGVPGAGVPGVGVPGVGVPGGKGVGVPGVGVP
GAGVPGVGVPGVGVPGVGVPGVGVPGVGVPGAGVPGVGVPGVGVPGGKGVGVPGVGVPGAGVPGVGVPGV
GVPGVGVPGVGVPGAGVPGVGVPGVGVPGGKGVGVPGVGVPGAGVPGVGVPGVGVPGVGVPGVGV
PGAGVPGVGVPGVGVPGGKGVGVPGVGVPGAGVPGVGVPGVGVPGVGVPGVGVPGAGVPGVGVPG
VGVPGGKGVGVPGVGVPGAGVPGVGVPGVGVPGVGVPGVGVPGAGVPGVGVPGVGVPGGKGVGVP
GVGVPGAGVPGVGVPGVGVPGVGVPGVGVPGAGVPGVGVPGVGVPGGKGVGVPGVGVPGAGVPGV
GVPGVGVPGVGVPGVGVPGAGVPGVGVPGVGVPGGKGCVLL

α subunit of FTase and GGTase-I

*(M)*AATEGVGESAPGGEPGQPEQPPPPPPPPPAQQPQEEEMAAEAGEAAASPMDDGFLSLDSPTYVLY
RDRAEWADIDPVPQNDGPSPVVQIIYSEKFRDVYDYFRAVLQRDERSERAFKLTRDAIELNAANYTVWHF
RRVLLRSLQKDLQEEMNYIIAIIEEQPKNYQVWHHRRVLVEWLKDPSQELEFIADILNQDAKNYHAWQHR
QWVIQEFRLWDNELQYVDQLLKEDVRNNSVWNQRHFVISNTTGYSDRAVLEREVQYTLEMIKLVPHNES
AWNYLKGILQDRGLSRYPNLLNQLLDLQPSHSSPYLIAFLVDIYEDMLENQCDNKEDILNKALELCEILAKE
KDTIRKEYWRYIGRSLQSKHSRESDIPASV

β subunit of FTase

*(M)*ASSSSFTYYCPPSSSPVWSEPLYSLRPEHARERLQDDSVETVTSIEQAKVEEKIQEVFSSYKFNHLVP
RLVLQREKHFHYLKRGLRQLTDAYECLDASRPWLCYWILHSLELLDEPIPQIVATDVCQFLELCQSPDGG
FGGGPGQYPHLAPTYAAVNALCIIGTEEAYNVINREKLLQYLYSLKQPDGSFLMHVGGEVDVRSAYCAAS
VASLTNIITPDLFEGTAEWIARCQNWEGGIGGVPGMEAHGGYTFCGLAALVILKKERSLNLKSLLQWVTSR
QMRFEGGFQGRCNKLVDGCYSFWQAGLLPLLHRALHAQGDPALSMSHWMFHQQALQEYILMCCQCPA
GGLLDKPGKSRDFYHTCYCLSGLSIAQHFGSGAMLHDVVMGVPENVLQPTHPVYNIGPDKVIQATTHFLQ
KPVPGFEECEDAVTSDPATD*QEEF*

The sequence highlighted in grey is translated from the RBS site that is proceeding the alpha subunit start codon in the translationally-coupled system (Figure 2a).

β subunit of GGTase-I

*(M)*AATEDDRLAGSGEGERLDFLRDRHVRFFQRCLQVLPERYSSLETSRLTIAFFALSGLDMLDSLDVVNK
DDIIEWIYSLQVLPTEDRSNLDRCGFRGSSYLGIPFNPSKNPGTAHPYDSGHIAMTYTGLSCLIILGDDLSR
VDKEACLAGLRALQLEDGSFCAVPEGSENDMRFVYCASCICYMLNNWSGMDMKKAISYIRRSMSYDNG
LAQGAGLESHGGSTFCGIASLCLMGKLEEVFSEKELNRIKRWCIMRQQNGYHGRPNKPVDTCYSFWVG
ATLKLLKIFQYTNFEKNRNYILSTQDRLVGGFAKWPDSHPDALHAYFGICGLSLMEESGICKVHPALNVST
RTSERLRDLHQSWKTKDSKQCSDNVHISS*QEEF*

4. Protein purification

We purified the proteins by optimizing a recently reported extraction method of ELP.[4] The method was optimized to apply for lipidated proteins as well as to reduce the isolation time to 40 min. The cells were pelleted by centrifuging at 21850 x g at 4 °C for 5 min. After decanting the supernatant, the cell pellet was resuspended in isopropanol (4 mL/g of the wet pellet). After thorough mixing by vortexing and bath sonication for 5 min, the cells were further mixed with isopropanol by rotating for 5 min at 25 °C. The protein was then separated in the supernatant by centrifuging at 15000 x g for 5 min at 25 °C. The protein was then precipitated by adding acetonitrile to the final composition of 70% (v/v). The solution was then centrifuged at 15000 x g for 5 min at 25 °C. After discarding the supernatant, the protein pellet was resuspended in 50% (v/v) ethanol in water. The suspension was centrifuged at 15000 x g, 25 °C for 5 min to remove any insoluble impurities. The protein was then analyzed and further purified by prep-HPLC. After organic extraction, the solution contained both unmodified and farnesylated ELPs. The lipidated product can be separated from the unmodified product by leveraging its lower transition temperature. In this paper, the farnesylated proteins were purified by preparative HPLC to ensure purity (>95%) for characterization studies. Reverse-phase HPLC (RP-HPLC) was performed with a Shimadzu HPLC system (Phenomenex Jupiter® 5 µm C18 300 Å, LC Column 250 × 10 mm, solvent A: H_2O + 0.1% TFA, solvent B: acetonitrile + 0.1% TFA). The percentage of the organic solvent in the mobile phase was increased from 0 to 90% over the course of 23 minutes. After HPLC purification, the organic solvent was removed by dialysis against water using SnakeSkin™ Dialysis Tubing (3500 MWCO, Thermo Scientific) overnight, followed by lyophilization. Lyophilized proteins were stored at -20 °C.

5. Molecular Characterization

5.1 Analytical HPLC

Analytical RP-HPLC was performed on a Shimadzu instrument using a Phenomenex Jupiter® 5 µm C18 300 Å, 250 × 4.6 mm LC Column with a mobile phase consisting of a gradient of acetonitrile in water containing 0.1% trifluoroacetic acid (Table S2) to analyze the proteins. The proteins were analyzed using a photodiode array detector at wavelengths between 190 and 230 nm.

5.2 Matrix-Assisted Laser Desorption/Ionization Time-of-Flight Mass Spectrometry (MALDI-TOF-MS)

MALDI-TOF-MS was conducted on Bruker microflex® LRF with a microScout ion source. A saturated solution of sinapinic acid in 50% acetonitrile was used as the matrix. The Samples were prepared by mixing 3 μL of 25 μM protein solutions with 7 μL of the matrix followed by serial dilution. These solutions were plated onto a sample plate and dried at room temperature. Apomyoglobin (M_W = 16,952.27 Da) was used as standard.

5.3 Trypsin Digestion

To identify the location of the farnesyl group, $(V/A)_{80}$-Fr were digested with trypsin, and the peptide fragments were analyzed using MALDI-TOF-MS. To set up the reaction, 9 μL of protein (100 μM) was added to 10 μL of 100 mM ammonium bicarbonate buffer (pH = 7.8) in an Eppendorf tube. The reaction was initiated by adding 1 μL trypsin (reconstituted as 5 μg/μL in 50 mM acetic acid) at 37 °C. After 3 h, the peptide fragments were analyzed by MALDI-TOF-MS. α-cyano-4-hydroxycinnamic acid was used as the matrix for the analysis of the trypsin-digested peptide fragments. The instrument was calibrated using adrenocorticotropic hormone (M_W = 2,464.1989 Da).

6. Turbidimetry assay

Temperature-triggered phase separation studies of the proteins were performed with an Agilent UV-Vis Spectrophotometer (Cary100) equipped with a Peltier temperature controller by measuring the absorbance of the solution at 350 nm. Four concentrations (3, 6, 10, and 12.5 μM in PBS) of proteins were analyzed by heating the solution at the rate of 1 °C/min from 15 to 65 °C. For reversibility studies, the protein solutions were then cooled to 15 °C at the same rate. The Transition temperature (T_t) was defined as the inflection point, i.e., the maximum of the first derivative, in the absorbance during the heating cycle. These data were fitted to the following model $T_t = -m \times \ln [\text{protein}] + T_c$ to derive critical transition temperature (T_c) and the concentration dependence of T_t (m), summarized in Table S3.

7. Supplementary Tables

Table S1. Plasmids used for expressing the proteins in this study.

Constructs	Vectors used for expression	Relevant features of the vector
V_{40}	pJMD[a]	Kan[r], pBR322 Ori, monocistronic T7 promotors
	pACYCDuet-1	Cm[r], p15A Ori, bicistronic T7 promotors
$(V/A)_{80}$	pJMD	Kan[r], pBR322 Ori, monocistronic T7 promotor
	pACYCDuet-1	Cm[r], p15A Ori, bicistronic T7 promotor
GGtase-I	pET23a or pETDuet-1[b]	Amp[r], pBR322 Ori, monocistronic T7 promotor
FTase-I	pET23a or pETDuet-1[b]	Amp[r], pBR322 Ori, monocistronic T7 promotor

[a] – pJMD is derived from pET24[1]; [b] – In the pETDuet-1 system, the production of α and β subunits from two mRNA transcripts (long and short) are not translationally coupled.[5] On the other hand, the pET23a vector produces a single mRNA transcript and translationally couples the production of two subunits. As discussed in the manuscript, the translationally coupled system substantially increased the production yield of active prenyltransferases by eliminating the accumulation of aggregation-prone subunits in the absence of the complementary stabilizing domain.

Table S2. The gradient mobile phase composition of analytical HPLC.

Time (min)	% (CH_3CN + 0.1% TFA)
0	0
5	40
12	50
22	53
23	90
28	90

Table S3. Critical transition temperature (T_c) and the concentration dependence of T_t derived from turbidity plots.

Construct	m (95% CI)[a]	T_c (°C, 95% CI)
V_{40}	-3.701 (-4.888 – -2.714)	51.6 (49.2 – 54.0)
V_{40}-Fr	-3.132 (-3.874 – -2.391)	36.4 (34.9 – 37.8)
$(V/A)_{80}$	-4.445 (-6.403 – -2.487)	61.0 (58.5 – 63.5)
$(V/A)_{80}$-Fr	-4.238 (-5.467 – -3.010)	47.5 (43.6 – 51.4)

[a] °C/ln (μM/μM). 95% confidence intervals are calculated from the linear regression analysis using Graphpad prism.

Table S4. The thermodynamic parameters for the LLPS of V_{40}, V_{40}-Fr, $(V/A)_{80}$, and $(V/A)_{80}$-Fr calculated from DSC curves.

Constructs	ΔH (mean ± SD, kcal/mol)[a]	ΔS (mean ± SD, cal/(mol.K))[a,b]
V_{40}	51.3 ± 5.2	701.8 ± 71.2
V_{40}-Fr	29.8 ± 1.5	418.9 ± 20.8
$(V/A)_{80}$	66.2 ± 3.9	878.6 ± 53.3
$(V/A)_{80}$-Fr	38.2 ± 0.3	521.3 ± 4.6

[a] – (n=2); [b] – calculated from $\Delta S = \Delta H/T_t$.

Table S5. Hydrodynamic size and polydispersity index derived from the analysis of autocorrelation functions using cumulants methods.

T (°C)	*V_{40}*		*V_{40}-Fr*		*$(V/A)_{80}$*		*$(V/A)_{80}$-Fr*	
	Z_{avg} (nm)[a]	PdI[a]	Z_{avg} (nm)	PdI	Z_{avg} (nm)	PdI	Z_{avg} (nm)	PdI
15	10±1	0.5±0.0	57±19	0.5±0.0	11±0.1	0.1±0.0	61±2	0.7±0.0
20	11±0.8	0.5±0.0	55±17	0.5±0.0	14±4	0.1±0.0	54±7	1±0.0
25	10±0.9	0.4±0.0	51±12	0.5±0.0	169±3	0.1±0.0	51±10	1±0.0
30	12±3	0.2±0.1	730±53	0.2±0.0	16±0.4	0.1±0.0	59±12	0.7±0.1
35	14±5	0.2±0.1	1599±56	0.1±0.0	17±2	0.1±0.0	65±16	0.7±0.1
40	245±102	0.3±0.1	1613±270	0.3±0.2	18±2	0.1±0.0	1589±1335	0.4±0.1
45	1146±339	0.2±0.0	1704±354	0.5±0.1	75±62	0.4±0.1	3853±1666	0.8±0.2
50	2468±772	0.5±0.1	1782±300	0.5±0.4	519±10	0.2±0.0	3988±2140	0.9±0.1
55	2799±840	0.5±0.3	1837±166	0.3±0.1	3350±1060	0.4±0.1	5303±2485	1±0.1
60	3316±50	0.4±0.2	1829±239	0.4±0.4	4587±484	0.7±0.2	5048±2359	1±0.2
65	2987±790	0.6±0.5	1882±288	0.2±0.1	4134±650	0.3±0.1	4988±2116	1±0.0

[a] – mean ± SD (2 independent samples, each measured in triplicate).

8. Supplementary Figures

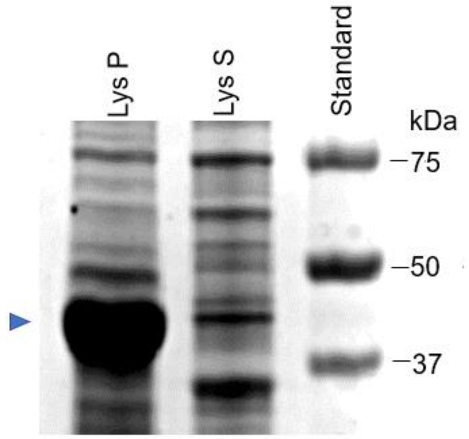

Figure S1. SDS-PAGE gel analysis of FTase expressed from a pETDuet-1 vector. The M_w of alpha and beta subunits are 44 and 48.6 kDa. FTase was predominantly present in the inclusion bodies (i.e., the insoluble pellet of cell lysate).

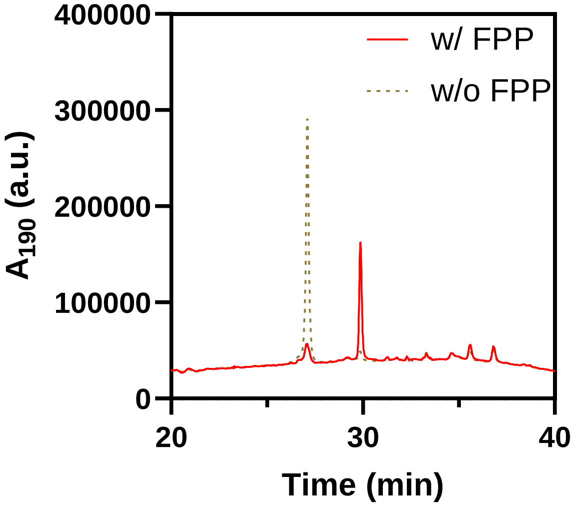

Figure S2. In vitro farnesylation of ELPs with the soluble transferase (produced as a translationally coupled heterodimer) indicates that the recombinantly expressed enzyme is active. The reaction mixture contained 3 µM of protein substrate (ELP-CVLS), 0.2 µM FTase, and 10 µM of farnesylpyrophosphate (FPP), and 5 mM TCEP in HEPPSO buffer (50 mM, pH = 7.8). The reaction mixture was incubated at room temperature for 16 h before analysis by RP-HPLC on a C_{18} column using a linear gradient of acetonitrile in water (0-90% over 40 min). The negative control lacked the FPP lipid donor.

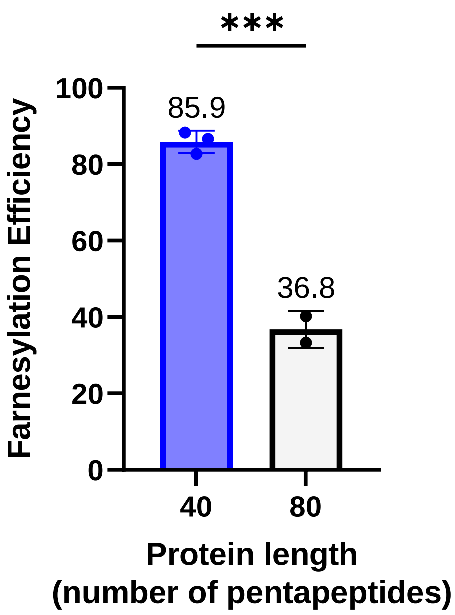

Figure S3. Farnesylation efficiency for coexpression of ELP-CVLL with GGTase-I (optimized substrate/enzyme combination for in vivo production of Fr-modified proteins). The farnesylation efficiency decreases with increasing the ELP length, unpaired two-tailed t-test, $p < .001$. Error bars are standard deviations of 2-3 independent replicates.

$$\text{Farnesylation efficiency} = AUC_{ELP\text{-}Fr} / (AUC_{ELP} + AUC_{ELP\text{-}Fr}) \times 100$$

AUC is the area under the curve, calculated by integrating the HPLC peak corresponding to non-lipidated and lipidated proteins. We and others have shown that model globular proteins (e.g., GFP) are efficiently farnesylated (33-85%, in vitro or in lysate) as long as their CaaX-motifs are accessible to prenyltransferases.[6-9] Thus, we hypothesized that the CaaX motif's accessibility and farnesylation efficiency are inversely correlated with ELP length (n, as a proxy for steric bulk). Consistent with this hypothesis, we observe that the farnesylation efficiency for 80-mer (~37%) is lower than 40-mer ELP (~86%), t (3) = 14.6, $p < .001$). We are cognizant that additional factors such as the flexibility of the linker connecting the CaaX motif to protein-of-interest can influence the farnesylation efficiency. Additional work is underway to illustrate how the length and hydrophilicity of the linker region influence the farnesylation efficiency.

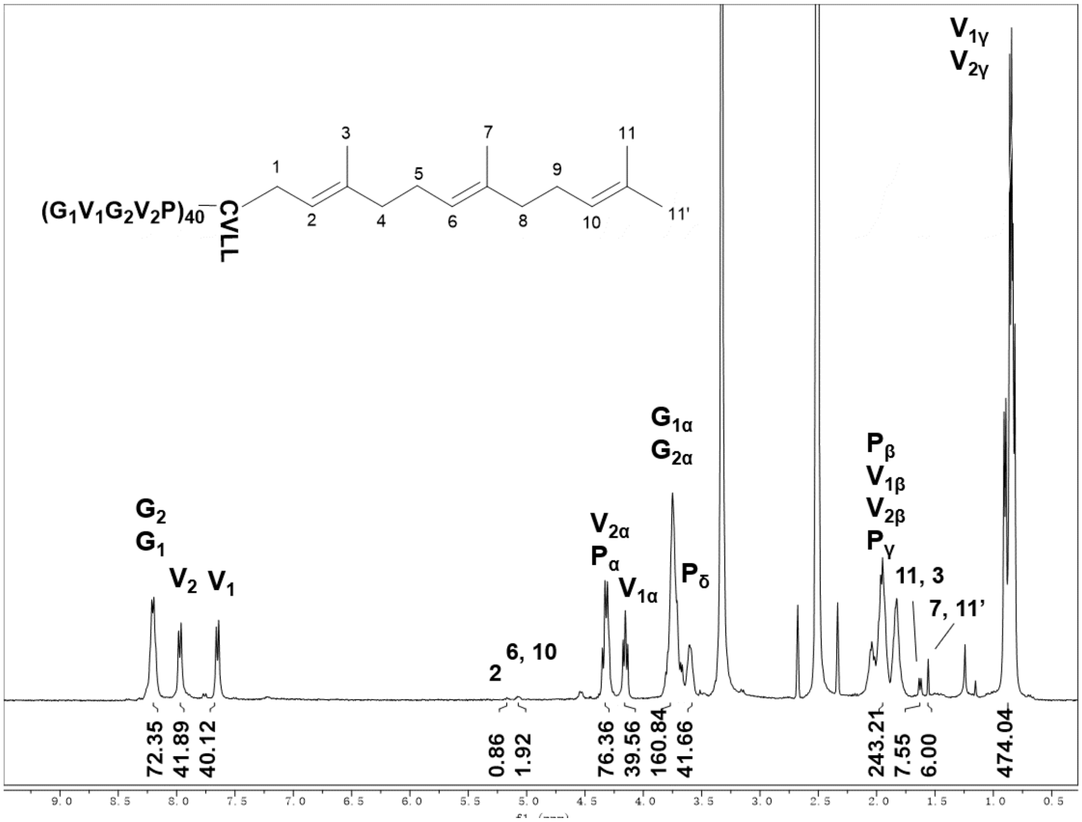

Figure S4. The assigned ^{1}H NMR spectra of V40-Fr in DMSO-d6 (2.5 ppm). The peak at 3.3 ppm is residual water.

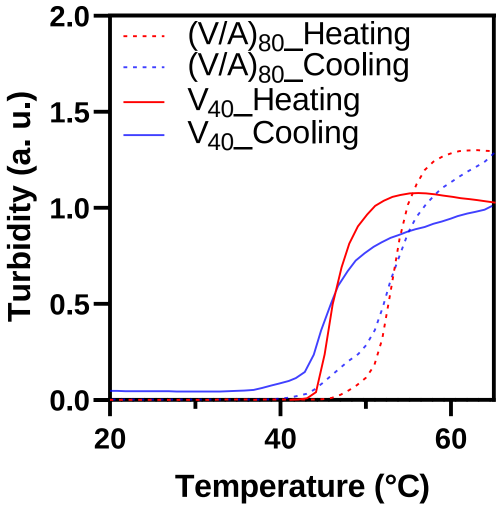

Figure S5. The temperature-triggered phase transition of V40 and (V/A)80 is reversible. [Protein] = 6 μM in PBS.

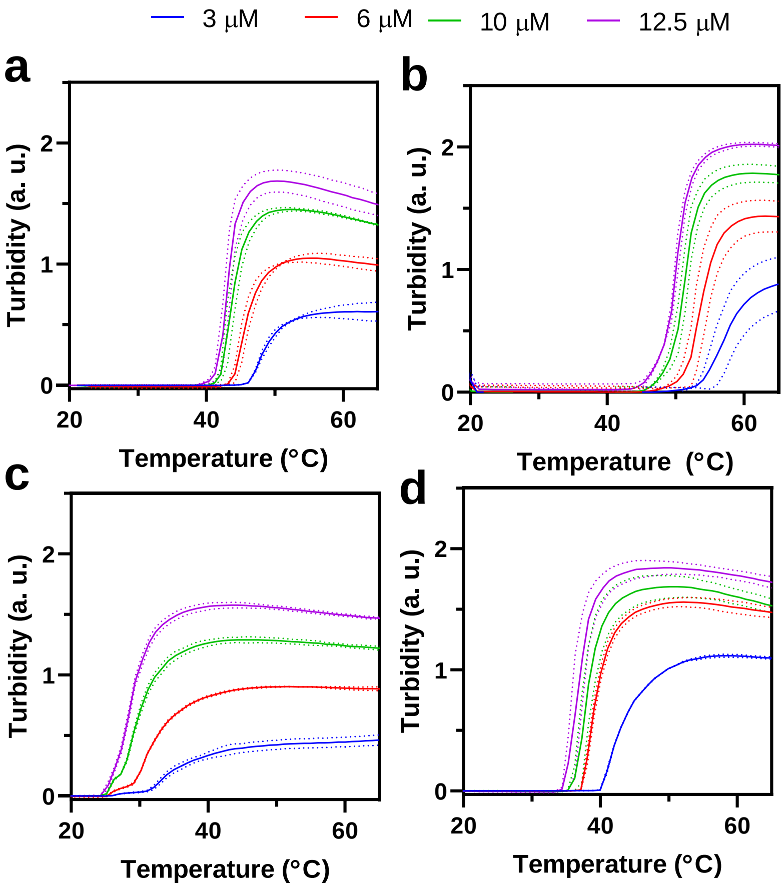

Figure S6. The concentration-dependent turbidimetry analysis of unmodified and farnesylated ELPs. **(a)** V_{40}, **(b)** $(V/A)_{80}$, **(c)** V_{40}-Fr, and **(d)** $(V/A)_{80}$-Fr. Dotted lines indicate the standard deviation of two independent measurements at each concentration.

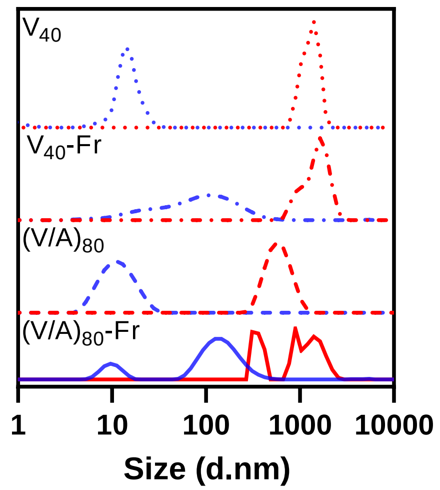

Figure S7. Size-intensity distribution of various constructs at 20 °C (blue) and 65 °C (red), below and above T_t respectively. All constructs form micron-size coacervates. [protein] = 6 µM in PBS.

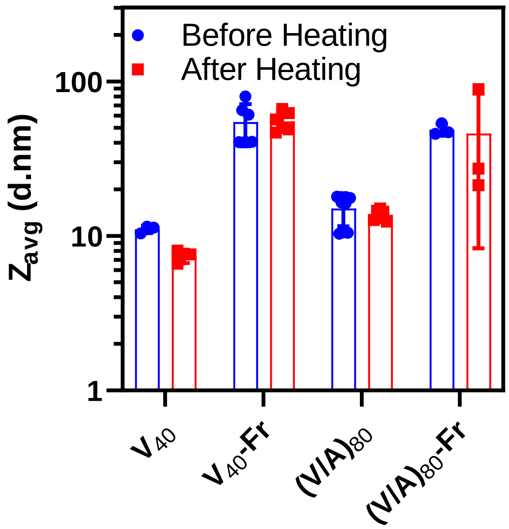

Figure S8. DLS confirms the reversible temperature-triggered phase-separation of unmodified and farnesylated proteins. The average hydrodynamic of proteins were measured at 20 °C (blue symbols and bars), and then after one cycle of heating and cooling (20 °C → 65 °C (above T_t) → 20 °C), red symbols and bars. No statistically significant differences between the average hydrodynamic radius of constructs before and after heating was observed (two-tailed nested t-test, e.g., for V_{40}-Fr t (10) = 0.07, p =.93). [protein] = 6 µM in PBS.

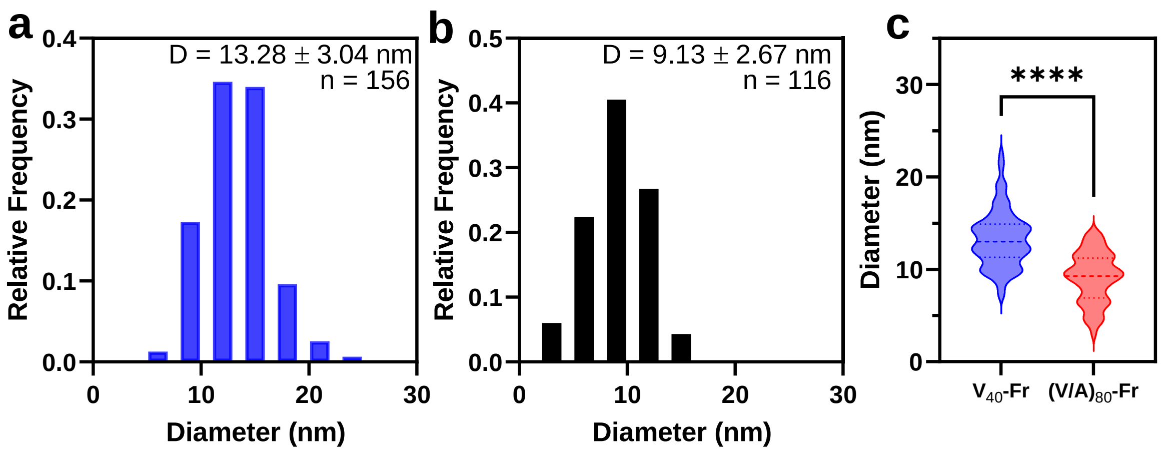

Figure S9. The size distribution of assemblies of V_{40}-Fr **(a)** and $(V/A)_{80}$-Fr **(b)** micelles obtained from the analysis of cryo-TEM images. **(c)** The violin plot shows the size distribution of particles' diameter. The horizontal dashed line denotes the median value, and dotted lines denote lower and upper quartiles. V_{40}-Fr formed larger assemblies compared to $(V/A)_{80}$-Fr (Two-tailed unpaired t-test, t (270) = 11.7, p <.0001).

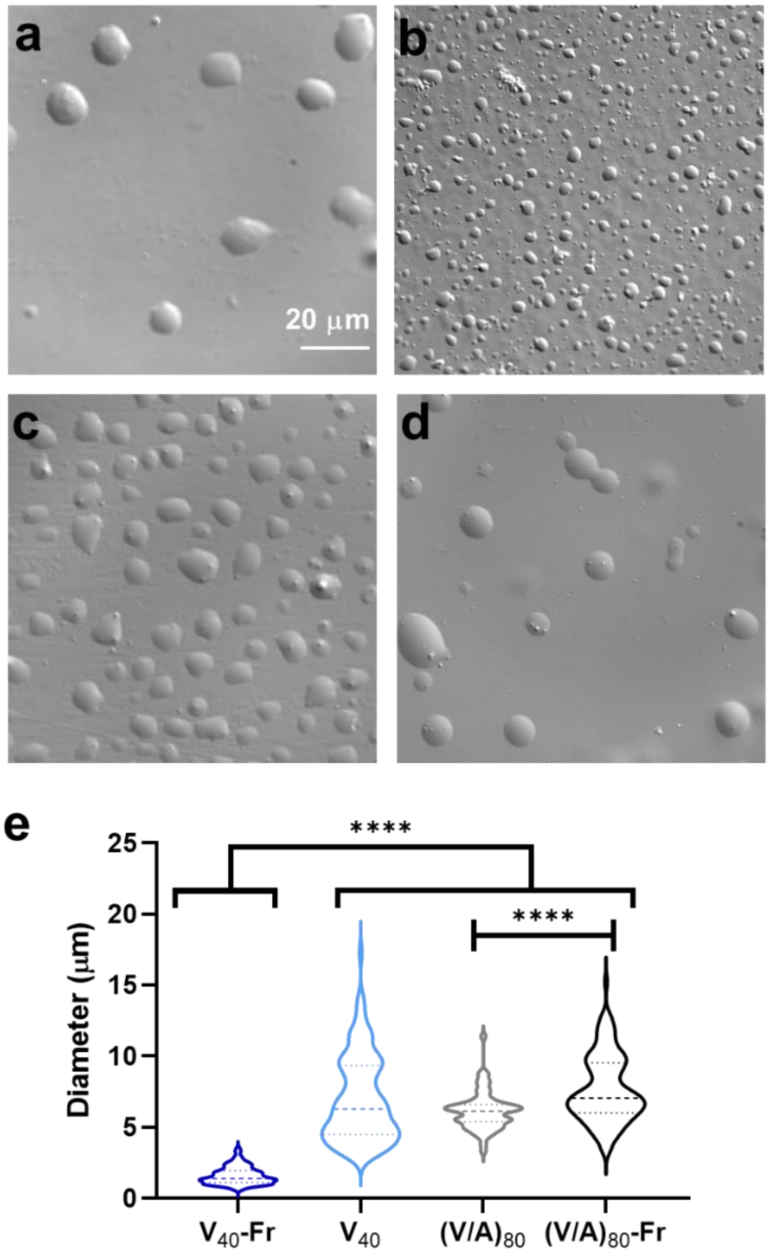

Figure S10. Characterization of protein coacervates formed by unmodified and farnesylated ELPs above their LCST using differential interference contrast (DIC) microscopy. **(a, b)** V_{40} and V_{40}-Fr; **(c, d)** $(V/A)_{80}$ and $(V/A)_{80}$-Fr. Both unmodified and farnesylated proteins undergo liquid-liquid phase separation and form spherical coacervates above their T_t. The apparent deformation (pinching) of some droplets is due to the adhesion to microscope slides. **(e)** The violin plot depicts the size (diameter)-distributions of coacervates. The horizontal dashed and dotted lines represent the median and quartiles (lower and upper). Consistent with DLS (Figure 5c), V_{40}-Fr coacervates (1.6 ± 0.6 μm) were noticeably smaller than V_{40} (6.9 ± 3.0 μm). However, $(V/A)_{80}$-Fr coacervates (7.7 ± 2.4 μm) were slightly larger than $(V/A)_{80,}$ 6.1 ± 1.3 μm. Measurements are reported as mean ± standard deviation. One-way ANOVA, $F(3,377) = 180$, p <0.0001, followed by Turkey's HSD test, ****: $p < .0001$.

9. References

1. McDaniel, J. R.; Mackay, J. A.; Quiroz, F. G.; Chilkoti, A., Recursive directional ligation by plasmid reconstruction allows rapid and seamless cloning of oligomeric genes. *Biomacromolecules* **2010,** *11* (4), 944-952.

2. Hartman, H. L.; Bowers, K. E.; Fierke, C. A., Lysine β311 of Protein Geranylgeranyltransferase Type I Partially Replaces Magnesium. *J. Biol. Chem.* **2004,** *279* (29), 30546-30553.

3. Zimmerman, K. K.; Scholten, J. D.; Huang, C.-c.; Fierke, C. A.; Hupe, D. J., High-level expression of rat farnesyl:protein transferase in Escherichia coli as a translationally coupled heterodimer. *Protein Expr. Purif.* **1998,** *14* (3), 395-402.

4. Sweet, C.; Aayush, A.; Readnour, L.; Solomon, K. V.; Thompson, D. H., Development of a Fast Organic Extraction–Precipitation Method for Improved Purification of Elastin-Like Polypeptides That Is Independent of Sequence and Molecular Weight. *Biomacromolecules* **2021,** *22* (5), 1990-1998.

5. Tolia, N. H.; Joshua-Tor, L., Strategies for protein coexpression in Escherichia coli. *Nat. Methods* **2006,** *3* (1), 55-64.

6. Wang, Y.; Kilic, O.; Csizmar, C. M.; Ashok, S.; Hougland, J. L.; Distefano, M. D.; Wagner, C. R., Engineering reversible cell–cell interactions using enzymatically lipidated chemically self-assembled nanorings. *Chem. Sci.* **2021,** *12* (1), 331-340.

7. Shala-Lawrence, A.; Blanden, M. J.; Krylova, S. M.; Gangopadhyay, S. A.; Beloborodov, S. S.; Hougland, J. L.; Krylov, S. N., Simultaneous Analysis of a Non-Lipidated Protein and Its Lipidated Counterpart: Enabling Quantitative Investigation of Protein Lipidation's Impact on Cellular Regulation. *Anal. Chem.* **2017,** *89* (24), 13502-13507.

8. Zhang, Y.; Blanden, M. J.; Sudheer, C.; Gangopadhyay, S. A.; Rashidian, M.; Hougland, J. L.; Distefano, M. D., Simultaneous Site-Specific Dual Protein Labeling Using Protein Prenyltransferases. *Bioconjug. Chem.* **2015,** *26* (12), 2542-2553.

9. Blanden, M. J.; Suazo, K. F.; Hildebrandt, E. R.; Hardgrove, D. S.; Patel, M.; Saunders, W. P.; Distefano, M. D.; Schmidt, W. K.; Hougland, J. L., Efficient farnesylation of an extended C-terminal $C(x)_3X$ sequence motif expands the scope of the prenylated proteome. *J. Biol. Chem.* **2018,** *293* (8), 2770-2785.
